# circUBAP2 exacerbates malignant capabilities of NSCLC by targeting KLF4 through miR-3182 modulation

**DOI:** 10.18632/aging.202745

**Published:** 2021-03-19

**Authors:** Guanying Zheng, Jianyuan Huang, Wenshu Chen, Peilin You, Yun Ding, Pengjie Tu

**Affiliations:** 1 Department of Pulmonary and Critical Care Medicine, Shengli Clinical Medical College of Fujian Medical University, Fujian Provincial Hospital, Fuzhou 350001, Fujian Province, China; 2 Department of Thoracic Surgery, Shengli Clinical Medical College of Fujian Medical University, Fujian Provincial Hospital, Fuzhou 350001, Fujian Province, China

**Keywords:** chemo-resistance, NSCLC, circUBAP2, therapeutic target

## Abstract

Chemo-resistance and refractoriness remain challenges for Non-small cell lung cancer (NSCLC) patients and the underlying molecular mechanisms haven’t been fully explained. In this study, we investigated the influence of circUBAP2 on the NSCLC tumor cells. This study might provide novel therapeutic targets for NSCLC treatment. Clinical samples and NSCLC cell lines were used to investigate circUBAP2 expressions and their impact on tumor cell chemo-resistance. CCK8 and transwell assays were conducted to explore the differences of NSCLC tumor proliferation and migration capabilities affected by circUBAP2. Dual-luciferase reporter gene assay was performed to explore the detailed molecular mechanism of circUBAP2 regulation network. circUBAP2 exhibited significantly elevated average level in our clinical samples of NSCLC, compared with normal tissues. CircUBAP2 level was positively correlated with disease stage and metastatic status. circUBAP2 significantly enhanced the migration, proliferation and chemo-resistance of NSCLC cell lines. Further experiments indicated that circUBAP2 promoted malignant biological behavior of NSCLC tumor cells by targeting KLF4 through modulating miR-3182 expression. Our study demonstrated for the first time that circUBAP2 played an important role exacerbating malignant capabilities of NSCLC. circUBAP2-miR3182-KLF4 regulative network demonstrated in this study could be a novel therapeutic target for future NSCLC treatment.

## INTRODUCTION

Lung cancer remains one of the deadliest malignancies worldwide [1]. Despite current advances in lung cancer diagnosis and treatment in the era of liquid biopsy and immunotherapy, disease refractoriness and distal organ metastasis have always been tough challenges for oncologists. For example, nearly 20% of advanced non-small cell lung cancer (NSCLC) patients suffered from disease refractoriness, which significantly shortened their survival [2, 3]. Therefore, it is of vital importance to understand the underlying detailed molecular mechanism in order to develop novel therapeutic interventions.

Circular RNAs (circRNAs) are a group of novel small RNAs that function as molecular sponges that modulate the expression of microRNAs through direct binding [4, 5]. Previous studies have indicated that circRNAs play an important role in the pathogenesis of several cancers including colorectal cancer [6], hepatocellular carcinoma [7], breast cancer [8] and gastric cancer [9]. Evidence from previous studies also provided an intriguing connection of tumor epithelial to mesenchymal transition (EMT) process to circRNAs biogenesis [10], which suggests potential roles of circRNAs in the metastasis of tumor cells.

circUBAP2 has been indicated as an important promoter in proliferation and chemo-resistant in multiple malignancies [11, 12]. However, it is still not known which gene modulates circUBAP2, especially in lung cancer. Therefore, in this study, we aimed to explore the molecular impact of circUBAP2 on lung cancer tumor cells and further delineate the detailed modulatory effects on its gene targets.

## RESULTS

### Characterization of circUBAP2

**Figure 1:**
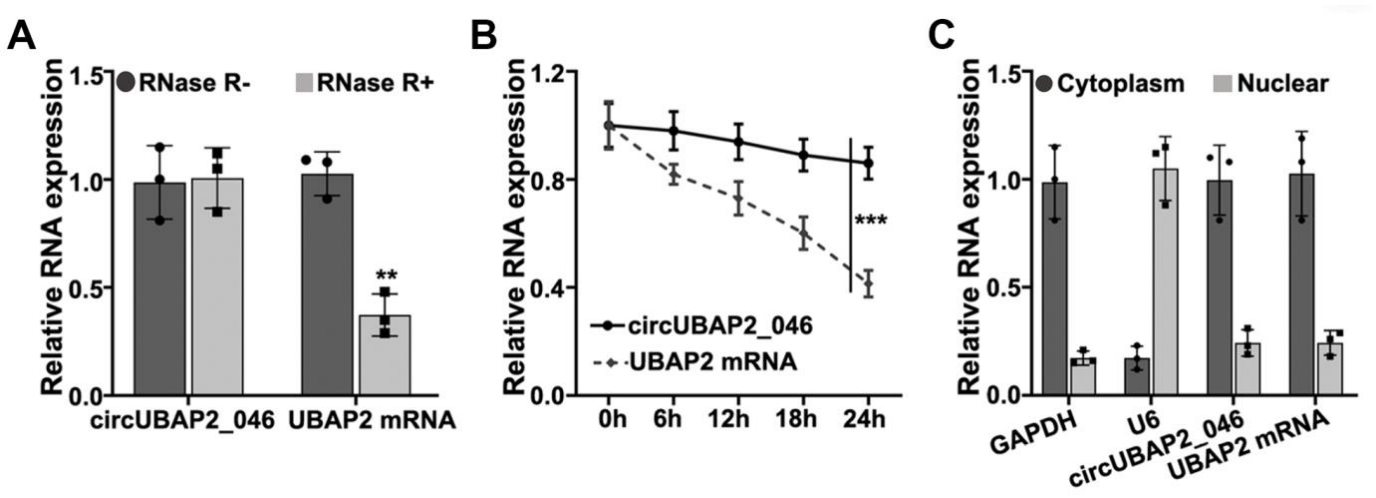
(**A**) RT-PCR detection of circUBAP2_046 and UBAP2 mRNA expression level in A549 cell line with/without treatment of RNase. (**B**) Detection of circUBAP2_046 and UBAP2 mRNA level by treatment of actinomycin-D after 0h, 6h, 12h, 18h, 24h. (**C**) RT-PCR analysis of circUBAP2_046 intracellular localization.

RNase treatment experiment indicated that circUBAP2 was highly stable, compared with UBAP2 mRNA (Figure 1A). Further experiments demonstrated that upon treatment with actinomycin D, which is a transcription inhibitor, circUBAP2 circular transcripts exhibited significantly higher stability compared with linear transcripts of UBAP2 mRNA (Figure 1B). In addition, we compared the expression level of UBPA2 mRNA and circUBAP2 in cytoplasm and nuclear. Results indicated both UBAP2 mRNA and circUBAP2 were significantly enriched in cytoplasm (Figure 1C).

### Expression of circUBAP2 in NSCLC samples

**Figure 2:**
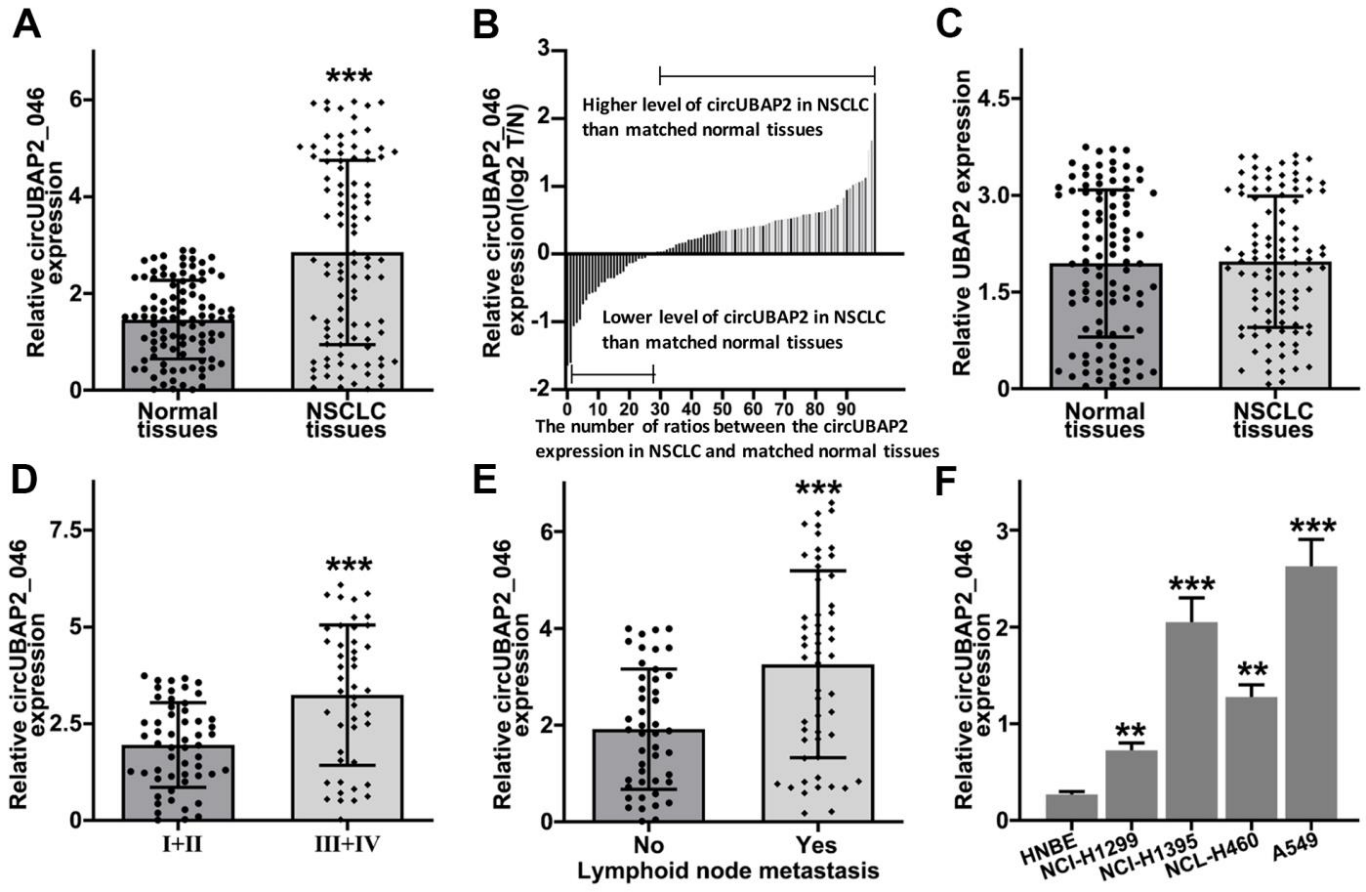
(**A**) RT-PCR detection of circUBAP2_046 expression level between NSCLC and matched normal tissue samples. (**B**) Relative circUBAP2_046 expression ratio distribution of NSCLC and matched normal tissue samples. (**C**) RT-PCR detection of UBAP2 expression level between NSCLC and matched normal tissue samples. (**D**) RT-PCR detection of circUBAP2_046 expression level between early stage (I/II) and advanced stage (III/IV) NSCLC samples. (**E**) RT-PCR detection of circUBAP2_046 expression level between NSCLC samples with/without lymph node metastasis. (**F**) RT-PCR detection of circUBAP2_046 expression level among different NSCLC cell lines (NCI-H1299, NCI-H1395, A549, NCL-H460) and human normal bronchial epithelial cell line HNBE.

In order to explore the role of circUBAP2 in NSCLC pathogenetic mechanism, we utilized RT-PCR method to detect circUBAP2 expression value in NSCLC and matched normal tissue samples. As a result, circUBAP2 exhibited significantly elevated average level compared with normal tissues (Figure 2A). We further compared the circUBAP2 expression level ratio of NSCLC and matched normal tissues. The results indicated that the majority of the tumor samples exhibited significantly higher level of circUBAP2 expression compared with matched normal tissues (Figure 2B). However, we did not observe significantly different level of UBAP2 mRNA expression level between NSCLC tumor samples and matched normal tissues (Figure 2C). Additionally, we further examined the expression of circUBAP2 level among NSCLC tumor samples of different stages. As a result, tumor samples from advanced-stage NSCLC patients (stage III-IV) showed significantly higher level of circUBAP2 expression level compared with tumor samples from early stages (stage I/II) (Figure 2D). As for patients with lymphoid node metastasis, their tumor samples also exhibited significantly higher level of circUBAP2, compared with those counterparts without lymphoid node metastasis (Figure 2E). Moreover, circUBAP2 expression levels among several NSCLC cell lines (NCI-H1299, NCI-H1395, A549, and NCL-H460) were significantly higher compared to normal human bronchial epithelial cell line HNBE (Figure 2F).

### Effects of circUBAP2 on biological behavior of NSCLC cell lines

**Figure 3:**
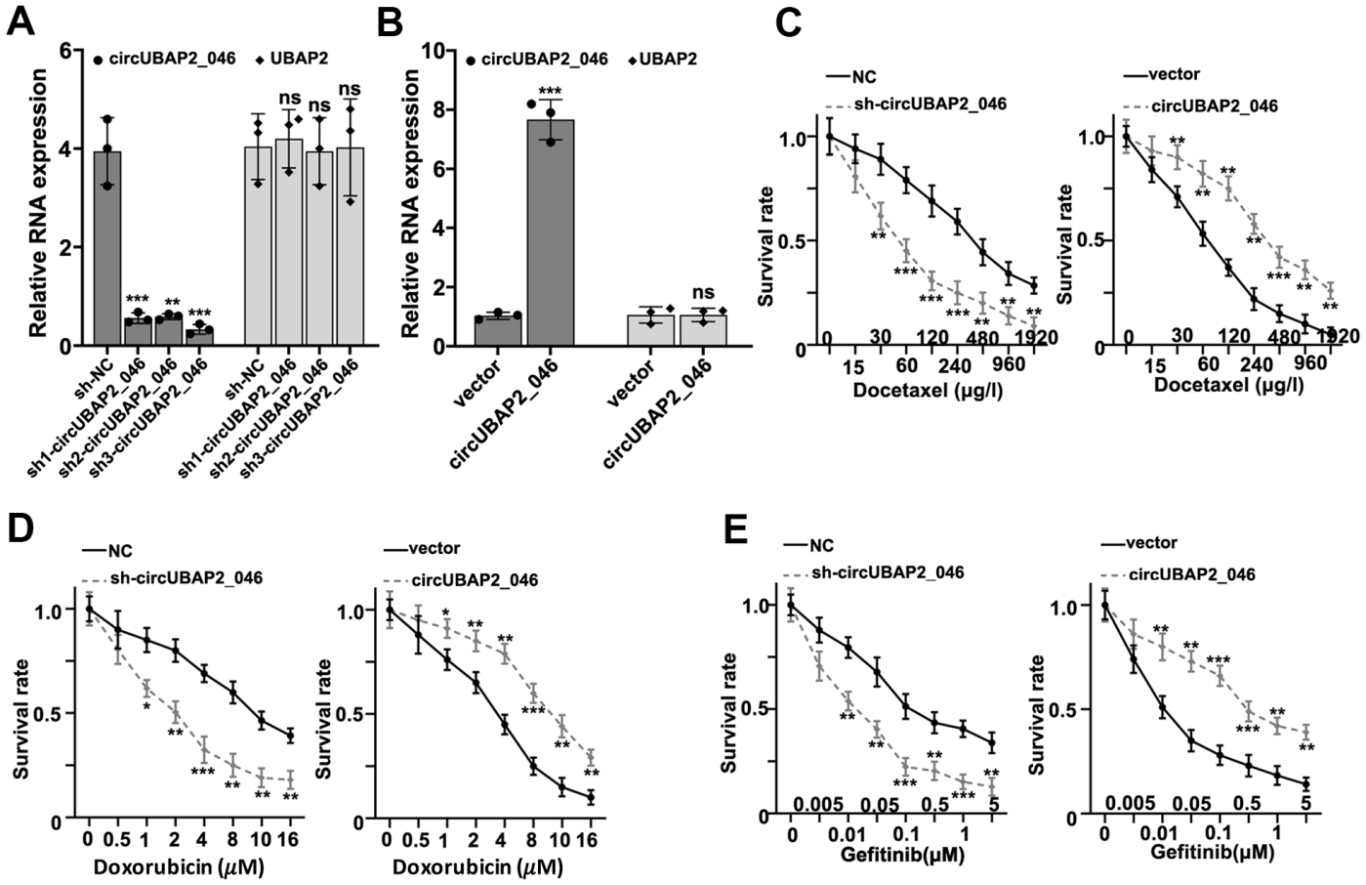
(**A**) RT-PCR detection of circUBAP2_046 and UBAP2 expression level in A549 cell line transfected by three different circUBAP2_046 specific shRNAs. (**B**) RT-PCR detection of circUBAP2_046 and UBAP2 expression level in NCI-H1299 cell line transfected with circUBAP2_046 over-expression vector. (**C**–**E**) Measurement of cell survival rate of cell groups (A549 cell group silenced by sh-circUBAP2_046, NCI-H1299 over-expressed by circUBAP2_046 and control group) treated with Docetaxel/Doxorubicin/Gefitinib of different concentrations.

**Figure 4:**
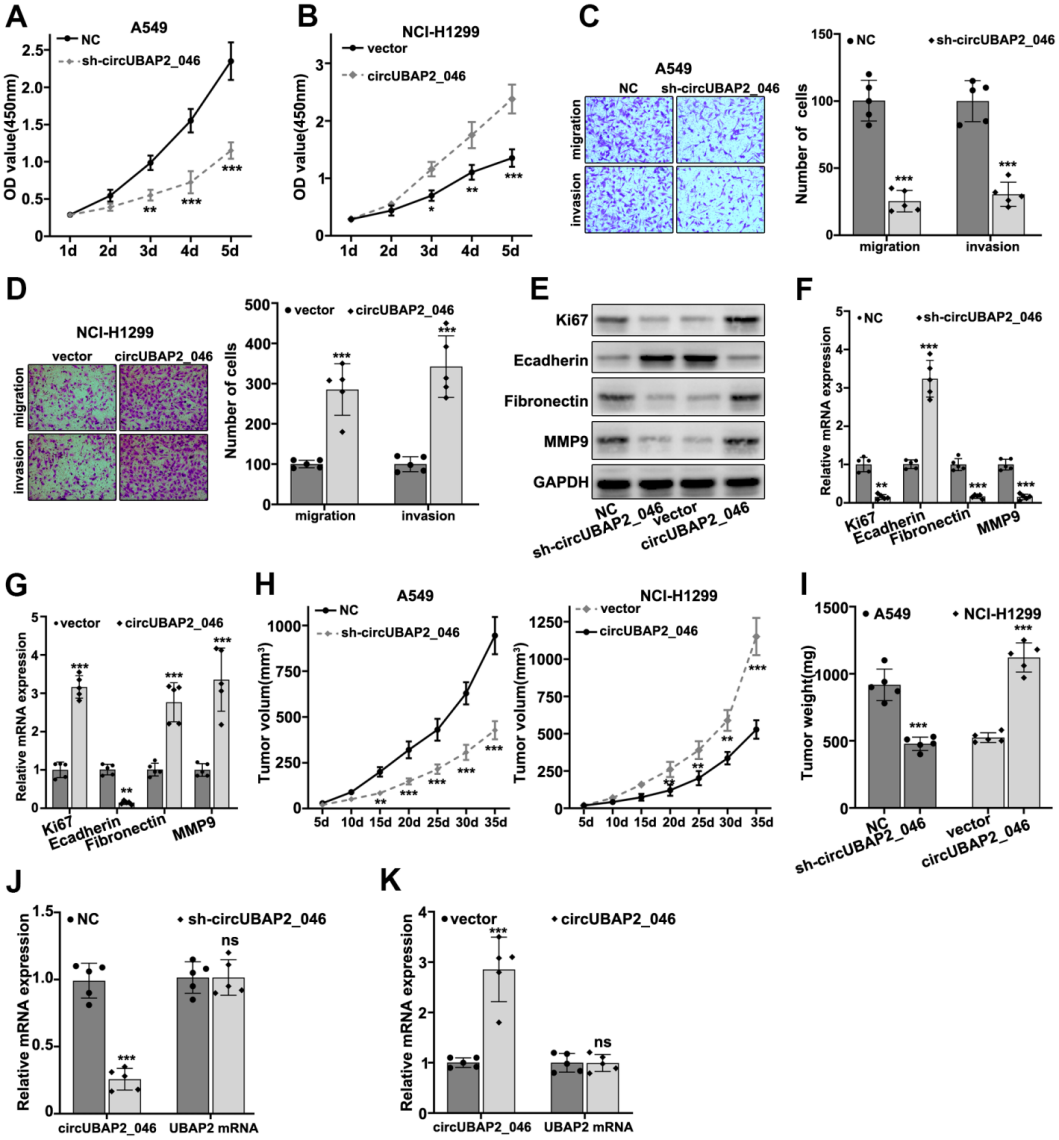
(**A**, **B**) CCK8 assay on A549 and NCI-H1299 cell line, which were transfected by sh-circUBAP2_046 or circUBAP2_046 overexpression vector. (**C**, **D**) Cell migration and invasion assay on A549 and NCI-H1299 cell transfected with sh-circUBAP2_046 or control. (**E**–**G**) WB and RT-PCR analysis on cellular proliferation and migration biomarkers including Ki67, E-Cadherin, Fibronectin and MMP9 in A549 /NCI-H1299 cell groups transfected with sh-circUBAP2_046 or circUBAP2_046 overexpression vector. (**H**, **I**) Impact of circUBAP2_046 silencing or over-expression on tumor growth and weight from *in vivo* A549/NCI-H1299 cell-derived xenograft models (5 tumors were measured for each group). (**J**, **K**) RT-PCR detection of circUBAP2_046 and UBAP2 mRNA expression level in xenograft tumor tissue of A549/NCI-H1299 cells transfected with sh-circUBAP2_046 or circUBAP2_046 overexpression vector (5 tumors were measured for each group).

To understand the role of circUBAP2 in biological behavior of NSCLC cell lines, we designed three different circUBAP2-specific shRNAs and circUBAP2 over-expression vector to modulate the expression level of circUBAP2. As shown in Figure 3A, 3B, all three shRNAs and circUBAP2 over-expression vector demonstrated significant suppressive and promoting effects on the expression level of circUBAP2 respectively, while they did not influence the mRNA expression level of UBAP2. Subsequently, we examined the impact of circUBAP2 expression level on cellular survivability under treatment of several chemotherapeutic agent including Docetaxel, Doxorubicin, and Gefitinib by transfecting sh-circUBAP2 and circUBAP2 overexpression vector into A549 and NCI-H1299 cells. As a result, cells transfected with sh-circUBAP2 exhibited significantly suppressed chemo-resistance when treated with escalated dosage of three different chemotherapeutic agents. On the other hand, cells transfected with circUBAP2 over expression vector demonstrated significantly enhanced survivability under treatment of chemotherapeutic agents compared with normal control (Figure 3C–3E). Next, we explored the impact of circUBAP2 modulation on the proliferative abilities of NSCLC cell lines by CCK8 assay. As described in Figure 4A, 4B, circUBAP2 over expression significantly increased the proliferate ability of NCI-H1299 cells while sh-circUBAP2 treated A549 cells suffered significantly suppressed proliferation compared with control group. Furthermore, transwell migration and invasion assay demonstrated that circUBAP2 expression elevation significantly boosted tumor cell invasive and migrated capabilities (Figure 4C, 4D). The above phenomena were also confirmed using Western blot (Figure 4E) and PCR analysis (Figure 4F, 4G) on specific biomarkers of cellular proliferation (Ki67), cellular migration (Fibronectin, MMP9) and adhesiveness (E-Cadherin). Our results suggested that circUBAP2 over expression was significantly related with enhanced cellular expression of Ki67, MMP9 and Fibronectin and suppressed expression of E-Cadherin. Moreover, we further utilized cell-derived xenograft (CDX) model to validate the effects of circUBAP2 expression modulation on tumor growth *in vivo*. Utilizing RT-PCR method, we detected significant differences in circUBAP2 expression level and comparable UBAP2 mRNA level in sh-circUBAP2 and circUBAP2 over expression groups, compared with controls (Figure 4J, 4K). As a result, circUBAP2 suppression by shRNA significantly hampered the growth of tumor volume from d5 - d35 post-inoculation. While circUBAP2 overexpression significantly accelerated tumor volume growth from d20-d35 post-inoculation (Figure 4H). Consistent results were shown by comparing tumor weight at d35 post-inoculation between circUBAP2 silencing/over expression group and control group, respectively (Figure 4I).

### circUBAP2 targeted KLF4 mRNA through modulation of miR-3182 expression

**Figure 5:**
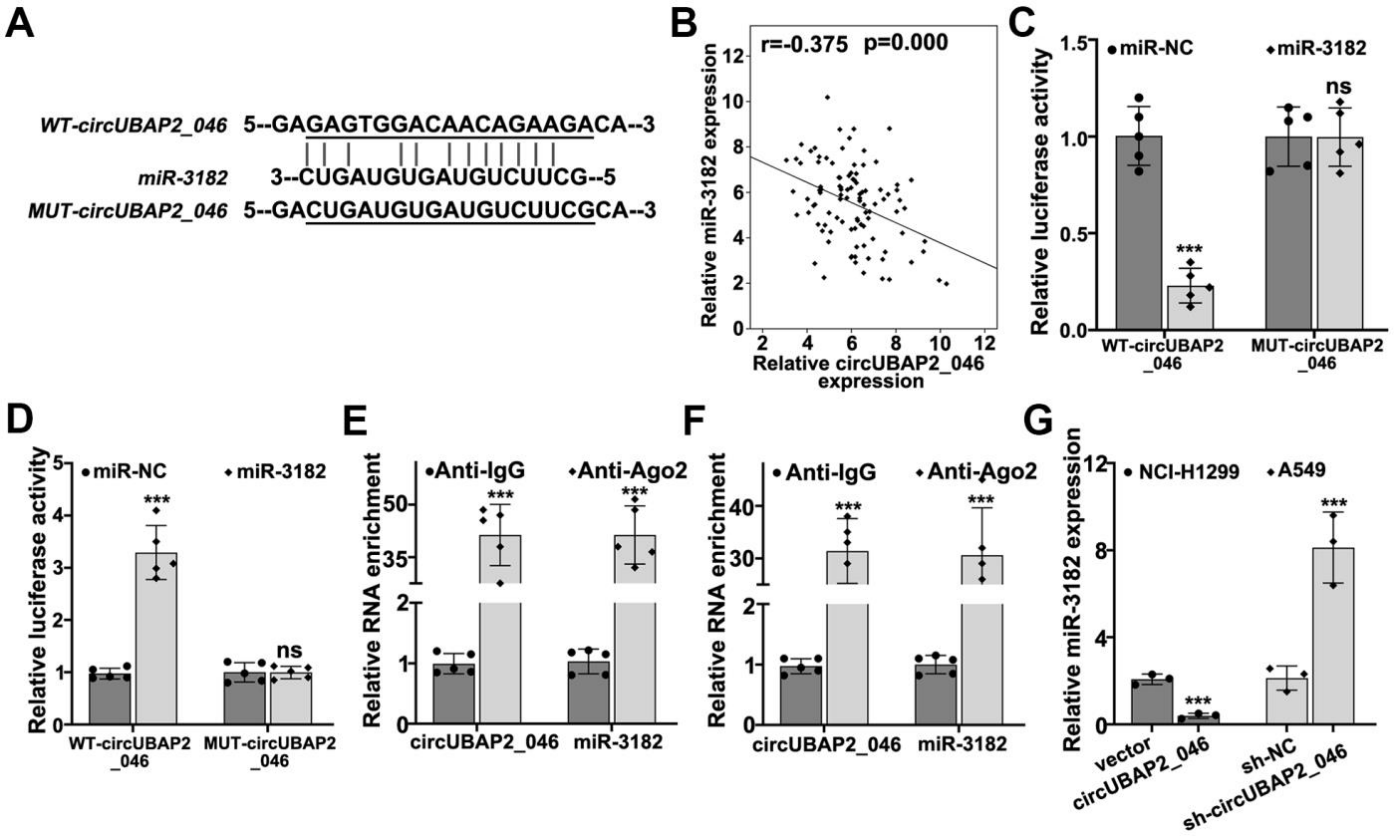
(**A**) Bioinformatic prediction on the binding sites of wild-type circUBAP2_046 or mutated circUBAP2_046 with miR-3182. (**B**) Correlations of mRNA expression value between miR-3182 and circUBAP2_046 in NSCLC lung cancer tissue samples. (**C**, **D**) Dual-luciferase reporter gene assay demonstrated the binding of circUBAP2_046 and miR-3182 in A549 and NCI-H1299 cell line models. (**E**, **F**) RNA immunoprecipitation assay demonstrated significantly enriched binding of circUBAP2_046 and miR-3182 with anti-Ago2 antibody compared with control IgG antibody in A549 and NCI-H1299 cell line models. (**G**) RT-PCR analysis about the modulative effects on miR-3182 expression by circUBAP2_046 silencing or overexpression.

Through interaction with RNA-induced silencing complex (RISC), circRNAs modulate miRNAs expression by acting as molecular sponges. In order to further explore the detailed molecular mechanism of circUBAP2, we performed bioinformatic analysis on binding prediction of circUBAP2. As depicted in Figure 5A, miR-3182 was predicted as potential target miRNA of circUBAP2. NSCLC clinical sample analysis also confirmed that miR-3182 expression level was negatively correlated with circUBAP2 expression (*P*<0.001). To further validate the potential interaction of miR-3182 and circUBAP2 *in vitro*, luciferase reporter gene assay was conducted and results indicated that miR-3182 demonstrated significantly decreased luciferase activity compared to control group when transfected with wild-type circUBAP2, while mutated circUBAP2 vectors had no influences on miR-3182 luciferase activity (Figure 5C, 5D). Through RNA immunoprecipitation (RIP) assay, we demonstrated that in A549 and NCI-H1299 cell lines, both circUBAP2 and miR-3182 bind with Ago (Argonaute) antibodies, which is the core protein of RISC, with significantly higher level of enrichment compared with those counterparts binding with control IgG (*P*<0.001) (Figure 5E, 5F). Moreover, miR-3182 expression was significantly suppressed by circUBAP2 over-expression and was significantly elevated by sh-circUBAP2 treatment (*P*<0.001).

### KLF4 is the target gene of miR-3182

**Figure 6:**
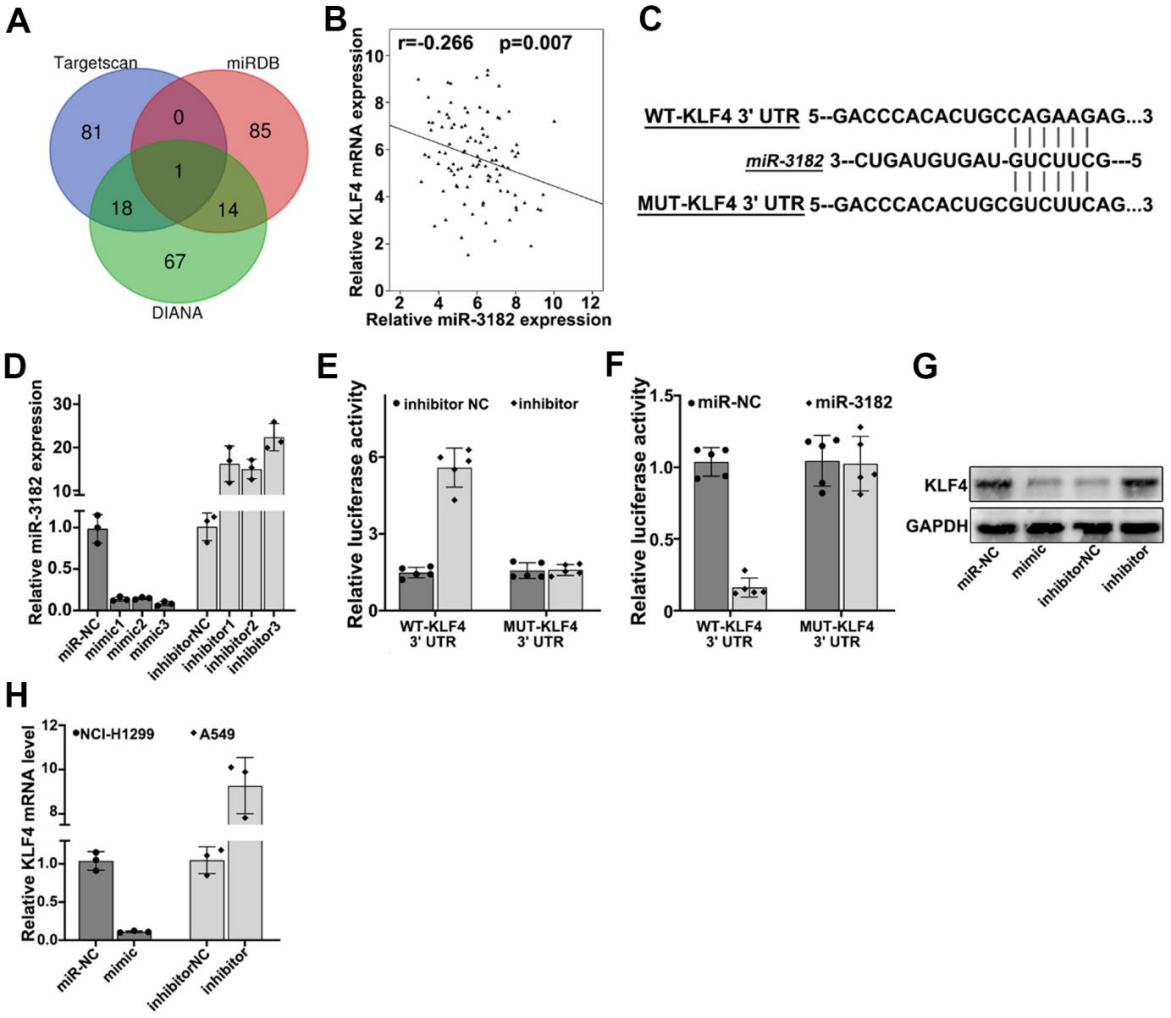
(**A**) Bioinformatic prediction of miR-3182 target genes via three major miRNA databases (Targetscan, miRDB, DIANA). (**B**) Correlation of mRNA expression of KLF4 and expression level of miR-3182 in NSCLC lung cancer tissue samples. (**C**) Bioinformatic prediction on the binding sites of miR-3182 with wild-type KLF4 and mutated KLF4 3’- UTR. (**D**) Effects of miR-3182 mimics and inhibitors modulative effects on miR-3182 expression value; (**E**, **F**) Dual-luciferase reporter gene assay on wild-type or mutated KLF4 expression influenced by miR-3182 inhibitor or miR-3182 over-expression. (**G**) Western-Blot analysis on the protein expression level of KLF4 influenced by miR-3182 mimic or inhibitor. (**H**) RT-PCR analysis on the mRNA expression level of KLF4 influenced by miR-3182 mimic or inhibitor.

To further understand the target gene regulatory function of miR-3182, bioinformatic prediction of miR-3182 targets genes were performed utilizing public databases including DIANA, TargetScan. miRDB, and KLF4 (Kruppel like factor 4) were shown as the target genes of miR-3182 for all three databases (Figure 6A). RT-PCR study on clinical samples indicated that KLF4 mRNA expression was negatively correlated with miR-3182 level (Figure 6B) (*P*=0.007). To further validate such modulatory effects of miR-3182 on KLF4 gene, miR-3182 binding site of KLF4 3’UTR region was predicted and vectors containing WT-KLF4-3’UTR and MUT-KLF4-3’UTR regions were generated respectively (Figure 6C). miR-3182 mimics and specific inhibitors were also built (Figure 6D). Dual-luciferase reporter gene assay was performed on A549 and NCI-H1299 cell lines transfected with WT-KLF4-3’UTR and MUT-KLF4-3’UTR, respectively. As a result, miR-3182 specific inhibitors significantly increased KLF4 expression level, while miR-3182 over-expression significantly downregulated KLF4 level. No apparent differences were detected in MUT-KLF4-3’UTR transfection group (Figure 6E, 6F). Consistent results were shown of miR-3182 mimics/inhibitors modulative function on KLF4 protein/mRNA level by Western-Blot and RT-PCR analysis (Figure 6G, 6H).

### circUBAP2 promoted proliferation and survivability of NSCLC cell lines by targeting KLF4 through miR-3182

**Figure 7:**
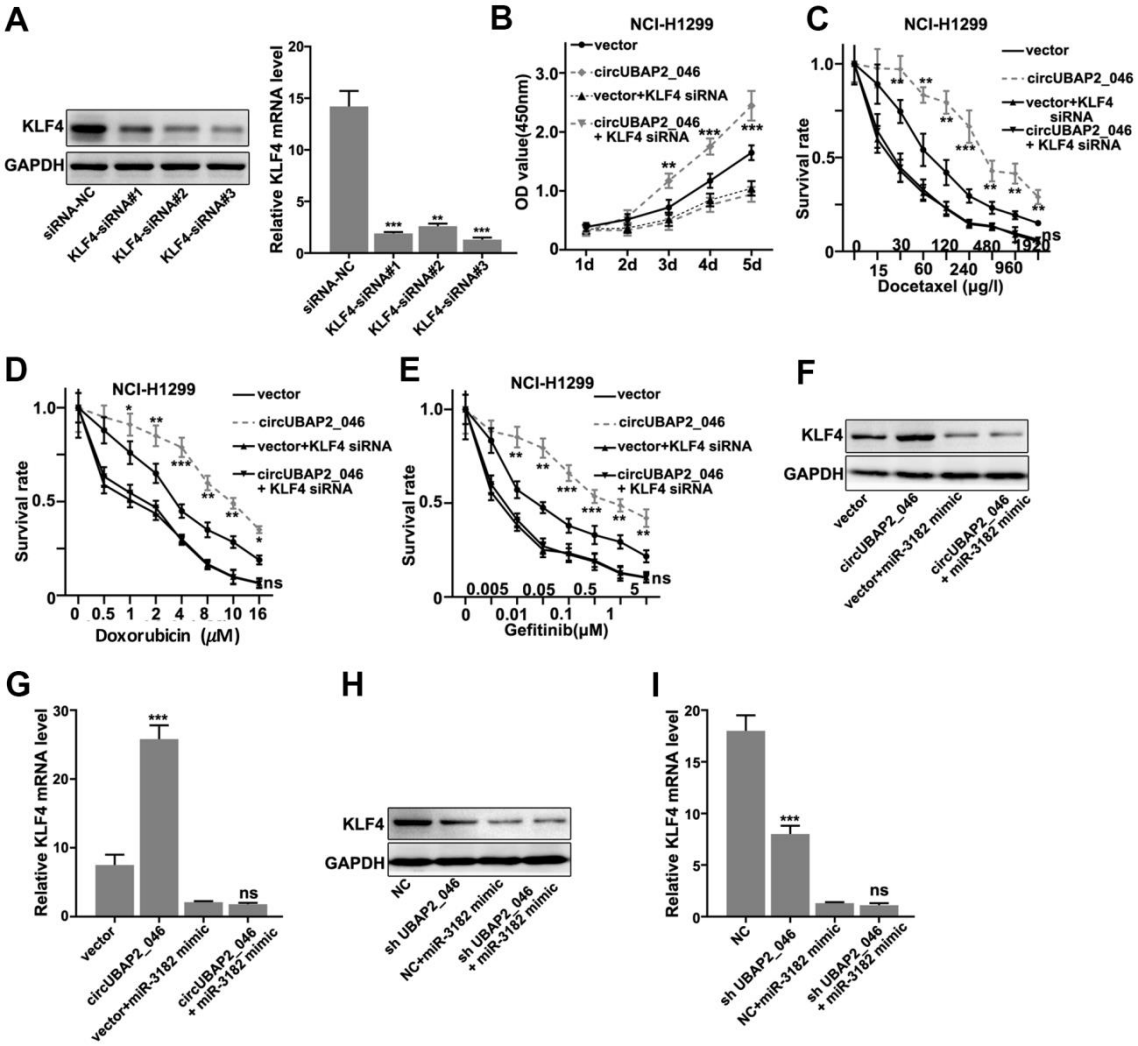
(**A**) Western-Blot and RT-PCR analysis on the siRNA inhibitory effects on the protein and mRNA expression level of KLF4. (**B**) CCK8 cell viability assay on NCI-H1299 cell line model transfected with circUBAP2_046 over-expression vector with/without KLF4 siRNA. (**C**–**E**) Cell survival assay on NCI-H1299 treated with escalated doses of Docetaxel. Cell line model was transfected with circUBAP2_046 over-expression vector with/without KLF4 siRNA. (**F**, **G**) Western-Blot and RT-PCR analysis on the KLF4 protein and mRNA expression value in A539 cell line model influenced by transfection of circUBAP2_046 over-expression vector with/without miR-3182 mimic. (**H**, **I**) Western-Blot and RT-PCR analysis on the KLF4 protein and mRNA expression value in NCI-H1299 cell line model influenced by transfection of circUBAP2_046 over-expression vector with/without miR-3182 mimic.

Moreover, in order to validate our findings, we designed KLF4-specific siRNAs and these siRNAs demonstrated significantly suppressive effects on KLF4 mRNA/protein expression (Figure 7A, 7B). CCK8 assay demonstrated KLF4 siRNAs abrogated circUBAP2 promoting effects on NCI-H1299 cell proliferation (Figure 7B). KLF4 siRNAs also abrogated the increased cellular survivability under chemo-agents treatment caused by circUBAP2 overexpression (Figure 7C–7E). Finally, through transfection of miR-3182 mimics and circUBAP2 overexpression vector in A549 and NCI-H1299 cell line, we detected significantly promoted expression of KLF4 mRNA and protein level in circUBAP2 overexpression group, while such effects were abrogated by co-transfection of miR-3182 mimics (Figure 7F–7I).

## DISCUSSION

NSCLC causes severe mortalities worldwide. Despite the fact that current standard chemoradiotherapy with/without surgery are effective in majority of patients, portions of patients suffered refractory disease and distal organ metastasis. Therefore, it is of great importance to unveil the underlying mechanism of lung cancer chemo-resistance. CircUBAP2 has been implicated to exhibit influences in tumor cell proliferation and survivability in multiple cancers [11, 12]. Our study confirmed that circUBAP2 level was not only higher in tumor compared with normal tissue, but was also positively correlated with disease severity. Therefore, once confirmed in larger scale of cohort, circUBAP2 could become a potential biomarker for accurate evaluation of lung cancer severity in clinical settings. Moreover, our research demonstrated via *in vitro* NSCLC cell line model that circUBAP2 played an important role in modulating the cellular chemo-resistance and migration. These results provided clues that novel target therapeutic interventions could be effective in refractory cases by targeting circUBAP2.

Our further analyses indicated that circUBAP2 modulated KLF4 expression by targeting miR-3182 through acting as molecular sponges. It has been suggested in several studies that miR-3182 was dysregulated in the pathogenesis of several malignancies [13, 14]. miR-3182 down-regulation was associated with up-regulation of genes involving several vital signaling pathways in carcinogenesis and tumor metastasis, including mTOR [15] and MMP2 [14]. miR- 3182 was also investigated as target of other circRNAs, such as linc00858 [13]. Our study provided further information on the complex regulation network of interaction between circRNAs and microRNAs in lung cancer cells, which requires further delicate experiments to quantify the impact of each element respectively.

Our results also indicated that KLF4 was the main target for circUBAP2 regulatory network. KLF4 has been confirmed to exert crucial functions in the physiological process of multiple organs, including intestine, eye, skin, bone and teeth [16–19]. Interestingly, KLF4 has been previously considered as tumor suppressor in several studies [20–23]. Recent research has indicated that KLF4 demonstrated anti-metastatic effects on NSCLC cells through SIRT6/Snail/KLF4 axis [21], and anti-proliferative effects through PLAC8/KLF4 axis [22]. However, our results provided seemly conflicting evidence that KLF4 overexpression caused by circUBAP2 dysregulation generated promoting influences on NSCLC proliferation and chemo-resistance. Other researchers also claimed that KLF4 could also serve as an oncogene under specific cellular conditions [24, 25]. Therefore, it is of value to further delineate the exact role of KLF4 on lung cancer pathogenesis and disease progression as well as metastasis. Moreover, other potentially affected gene pathways by circUBAP2 and miR-3182 should also be further investigated to fully understand the impact of circUBAP2 dysregulation on NSCLC patients.

It is worth mentioning that our study was generally based on limited number of clinical samples and *in vitro* cell line models, future studies of expanded clinical cohorts and *in vivo* animal models are required to further validate our findings in this study.

Our study demonstrated for the first time that circUBAP2 played an important role in promoting proliferation, invasion and chemo-resistance of NSCLC tumor cells. circUBAP2 might be informative biomarker for NSCLC clinical severity and metastasis prediction. circUBAP2-miR3182-KLF4 regulative network demonstrated in this study could be a novel therapeutic target for future NSCLC treatment.

## MATERIALS AND METHODS

### Patient recruitment and sample collection

Our clinical cohort was recruited from patients diagnosed of NSCLC in cancer center from Aug 2018 to Jun 2019. A total of 60 patients’ tumor biopsy samples and adjacent normal tissues were retrieved during surgery. No prior treatments were conducted before surgery. Tissues were stored using liquid nitrogen immediately after resection for subsequent experiments. The study was approved by Ethical Committee of Fujian Provincial Hospital. Informed consent was obtained for all patients enrolled in this study.

### Cell line culturing

NSCLC cell line NCI-H1299, NCI-H1395, A549, NCL-H460 were purchased from American Type Culture Collection (ATCC; Manassas, VA, USA). Cells were cultured with RPMI-1640 medium plus 10% fetal bovine serum (FBS; Hyclone, South Logan, UT, USA), and 100 IU/mL penicillin combined with 100 μg/mL streptomycin (Invitrogen, Carlsbad, CA, USA), under environment of 37° C, 5% CO_2_. The mycoplasma in all cell lines was tested using PCR gel electrophoresis (Supplementary Figure 1). Morphology images of cell lines were listed in the Supplementary Figure 2.

### Cell transfection

NCI-H1299 and A54-9 cells were placed into 8-well plates at the density of 1x10^6^ cells per well. Lipofectamine 2000 (Invitrogen, Carlsbad, CA, USA) was used for transfection of vectors including circUBAP2_46 overexpression plasmid, miR-3182 mimics and inhibitor, sh-CircUBAP2_46, KLF4 siRNAs according to the instructions of manufacturer.

### Real time qRT-PCR quantification

Total RNA was extracted using TRIzol agent (Invitrogen, Thermo Fisher Scientific, Inc) according to the standardized protocol. cDNA was generated by reverse transcription for subsequent qRT-PCR experiments. qRT-PCR reaction condition was set as 94° C for 30s, 55° C for 30s, 72° C for 90s, with total cycles of 40. Primers used in this study include: UBAP2 forward 5’-CCCAGTGCCCTTTCCTAGAA -3; reverse 5’-TACAGTGGAGAAGGGGCTTG-3’; circUBAP2 forward 5’- AGCCTAGAGCCAACTCCTTTG -3; reverse 5’- TCAGGTTGAGATTTGAAGTCAAGA-3’; U6 forward, 5’-CTCGCTTCGGCAGCACAT-3’; reverse, 5’-AACGCTTCACGAATTTGCGT-3; miR-3182 forward, 5’-GGTGAGGCAGTAGATTGAATCTC-3’; reverse, 5’-CTCAACTGGTGTCGTGGAGTC-3’; Actin forward, 5’-GTCCACCGCAAATGCTTCTA-3’; reverse, 5’-TGCTGTCACCTTCACCGTTC-3’.

### Western blot

Total protein from the samples were extracted with RIPA agent (Beyotime, Shanghai, China) and quantified by BCA protein assay kit. Protein samples were electrophoresed on polyacrylamide gels and then transferred to polyvinylidene difluoride (PVDF) membranes (Millipore, Billerica, MA, USA). The membranes were incubated with primary antibodies at 4° C overnight. Ki67 (#ab16667, 1:1000, Abcam, Cambridge, UK), E-Cadherin (#3195, 1:1500, Cell Signaling Technology, Danvers, MA, USA), Fibronectin (#26836, 1:1000, Cell Signaling Technology), MMP9 (#13667, 1:1000, Cell Signaling Technology), KLF4 (#12173, 1:1000, Cell Signaling Technology), GAPDH (#5174, 1:1000, Cell Signaling Technology). After rinsing with the Tris-Buffered Salin and Tween buffer solution (TBST; Sigma-Aldrich, St. Louis, MO, USA), they were incubated with the secondary antibody (#ab205718, 1:2000, Abcam, Cambridge, UK) at room temperature for 1 h. Chemiluminescence was used to expose the protein bands on the membrane.

### Cell proliferation CCK8 assay

Cells were inoculated into 96-well plates at a density of 1×10^3^ cells/well. 10 μL of Cell Counting Kit-8 (CCK-8; Dojindo Molecular Technologies, Kumamoto, Japan) solution was added to each well after 1d, 2d, 3d, 4d and 5d, respectively, followed by incubation at 37° C for 1 h. The absorbance was recorded by a microplate reader of each well at 450 nm.

### Cell survivability and migration assay

1×10^6^ Cells for each group were treated with escalated dosage of Docetaxel, Doxorubicin Gefitinib for 4h, and then cells were washed with cold PBS, and then incubated in 75% ethanol at -20° C overnight. Cells were stained with 10 μL propidium iodide (PI) and Annexin V-FITC (Thermo Fisher Scientific) for 20 min at room temperature, and apoptotic cells were analyzed by flow cytometry (FACScan™, BD Biosciences, Franklin Lakes, NJ, USA). 5×10^4^ transfected cells were seeded into the upper chamber (8-μm) (Corning, Lowell, MA, USA). The medium with 10% fetal bovine serum (FBS; Gibco, Grand Island, NY, USA) was added to the bottom chamber. The cells were incubated under 37° C, 5% CO_2_ condition for 48 h. Penetrating cells were fixed in 70% ethanol for 30 min and stained with 0.1% crystal violet for 10 min. Five randomly selected fields per sample was counted for the number of penetrating cells.

### RNA-immunoprecipitation

Nuclear proteins of cells were extracted according to the manufacturers’ instructions. 10% of the total nuclear protein was used as input control. The remaining protein were incubated with anti-IgG and anti-Ago2 antibodies at 4° C for 3 h, and subsequently incubated with protein A/G plus-agarose at 4° C overnight. The proteins were then centrifuged at 4° C, 2000 r/min for 1 min. The precipitate was then re-suspended in NETN100. 10% of precipitate, protein level was quantified using input control and IgG sample. Meanwhile, the remaining samples were used for RNA isolation, purification and identification.

### Dual-luciferase reporter gene assay

Cells were digested with trypsin and inoculated into 24-well plates prior to transfection. Transfection reagents were prepared as described below: Tube A: circUBAP2-WT plasmid and circUBAP2-MUT plasmid or KLF4-WT/MUT plasmid were mixed with culture medium; Tube B: miR-3182-WT plasmid and miR-NC plasmid were mixed with culture medium; Tube C: transfection reagent was mixed with culture medium. Tube C mixture was separately added into Tube A and B. Mixtures in Tube A and B were added in each well and incubated for 48 h. Transfection efficiency was observed by a fluorescence microscope (Leica, Wetzlar, Germany).

### *In vivo* assay

Animal model experiments were approved by the Animal Care and Use Committees at Fujian Provincial Hospital. 2×10^6^ A549/NCI-H1299 cells in 100 μl PBS were injected into the right flank of 5-weeks old female BALB/c nude mice. Tumor volumes and weights were measured 5d, 10d, 15d, 20d, 25d, 30d, 35d post-inoculation and animal sacrifice.

### Statistical analysis

Statistical Product and Service Solutions (SPSS) 22.0 (Chicago, IL, USA) was used for all statistical analysis. GraphPad Prism (Version X; La Jolla, CA, USA) was introduced for figure processing. Experimental data were expressed as mean ± standard deviation (SD) (x– ± s). Standard t-test was used to compare the differences between the two groups. Gene correlation was compared using Person analysis. Chi-square test was used for analyzing classification data. p<0.05 was considered statistically significant.

### Ethics approval and consent to participate

The study was approved by Ethical Committee of Fujian Provincial Hospital (Approval number: 2019-36). Informed consent was obtained for all patients enrolled in this study.

## Supplementary Material

Supplementary Figures
