# Correction for: circUBAP2 exacerbates malignant capabilities of NSCLC by targeting KLF4 through miR-3182 modulation

**DOI:** 10.18632/aging.203928

**Published:** 2022-03-14

**Authors:** Guanying Zheng, Jianyuan Huang, Wenshu Chen, Peilin You, Yun Ding, Pengjie Tu

**Affiliations:** 1 Department of Pulmonary and Critical Care Medicine, Shengli Clinical Medical College of Fujian Medical University, Fujian Provincial Hospital, Fuzhou 350001, Fujian Province, China; 2 Department of Thoracic Surgery, Shengli Clinical Medical College of Fujian Medical University, Fujian Provincial Hospital, Fuzhou 350001, Fujian Province, China

**Keywords:** chemo-resistance, NSCLC, circUBAP2, therapeutic target

Original article: Aging. 2021; 13:11083–11095.  . https://doi.org/10.18632/aging.202745

**This article has been corrected:** A small overlap was found in **Figure 4C** between the A549 cells negative control (NC) migration and invasion panels. This was the result of misfiling the data. The new Figure 4C contains a new invasion panel for the A549 cells NC. In **Figure 4D,** the invasion image for NCI-H1299 cells transfected with circUBAP2_046 was mixed up with the migration image. The new Figure 4D contains new invasion panels for NCI-H1299 cells transfected with circUBAP2_046 or NC vector. To correct **Figure 4C** and **D,** the authors used representative images from the original sets of experiments. These corrections do not affect the results or conclusions of this work. The authors would like to apologize for any inconvenience related to this mistake.

New **Figure 4** is presented below.

**Figure 4:**
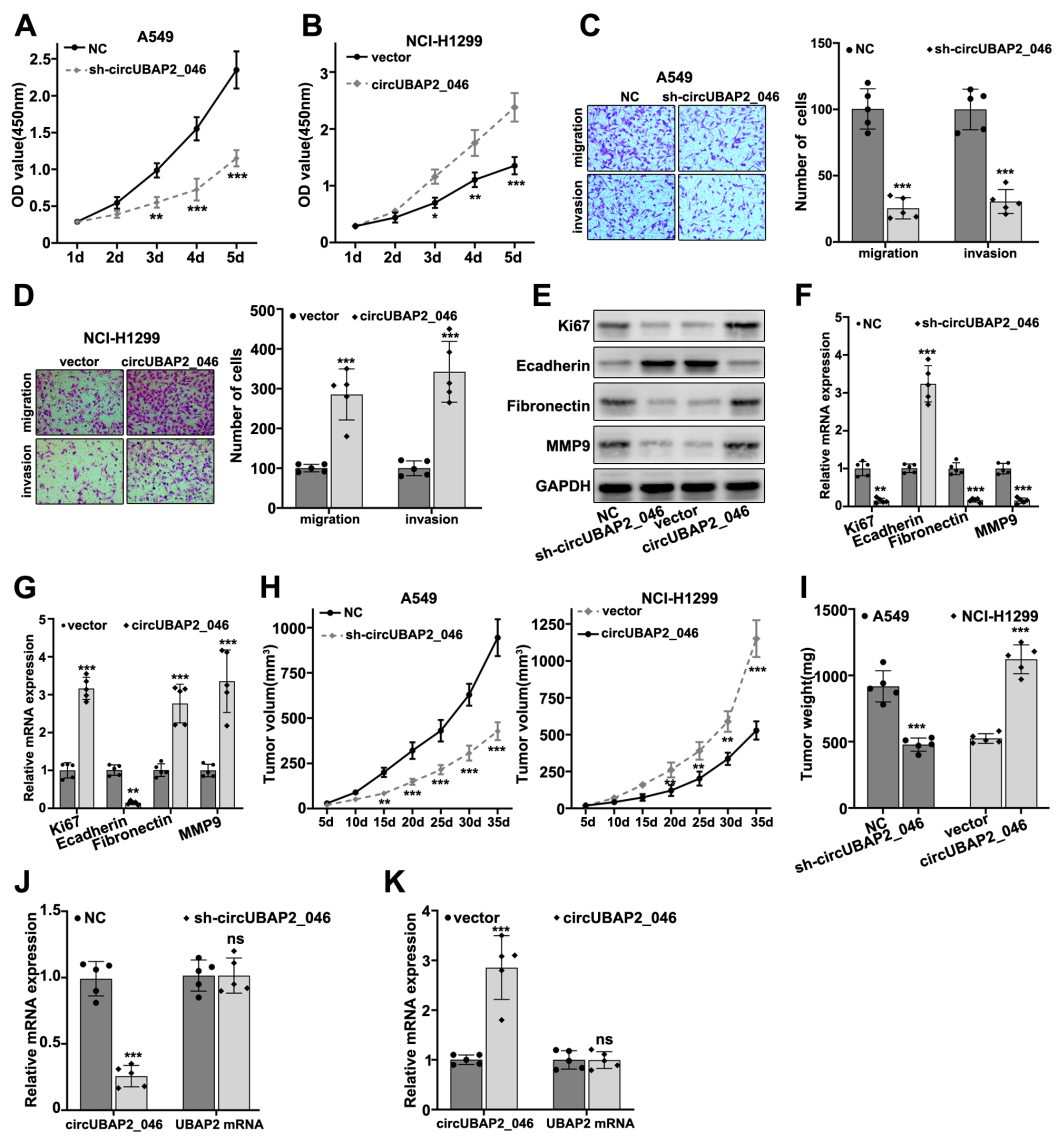
(**A**, **B**) CCK8 assay on A549 and NCI-H1299 cell line, which were transfected by sh-circUBAP2_046 or circUBAP2_046 overexpression vector. (**C**, **D**) Cell migration and invasion assay on A549 and NCI-H1299 cell transfected with sh-circUBAP2_046 or control. (**E**–**G**) WB and RT-PCR analysis on cellular proliferation and migration biomarkers including Ki67, E-Cadherin, Fibronectin and MMP9 in A549 /NCI-H1299 cell groups transfected with sh-circUBAP2_046 or circUBAP2_046 overexpression vector. (**H**, **I**) Impact of circUBAP2_046 silencing or over-expression on tumor growth and weight from *in vivo*A549/NCI-H1299 cell-derived xenograft models (5 tumors were measured for each group). (**J**, **K**) RT-PCR detection of circUBAP2_046 and UBAP2 mRNA expression level in xenograft tumor tissue of A549/NCI-H1299 cells transfected with sh-circUBAP2_046 or circUBAP2_046 overexpression vector (5 tumors were measured for each group).

