# Supplementary material for: circUBAP2 exacerbates malignant capabilities of NSCLC by targeting KLF4 through miR-3182 modulation
Source: Aging (Albany NY). 2021 Mar 19;13(8):11083–95. doi: 10.18632/aging.202745 (PMC8109095; doi:10.18632/aging.202745)
Supplement: Supplementary Figures [file aging-13-202745-s001.pdf]

SUPPLEMENTARY FIGURES

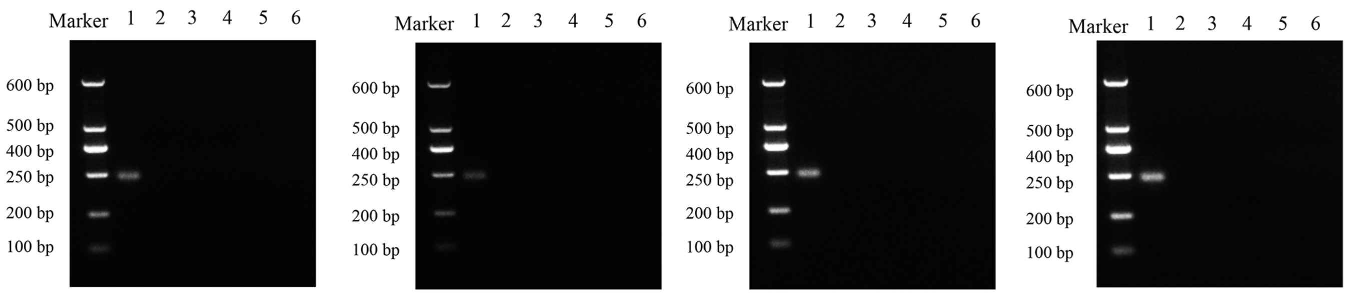

Supplementary Figure 1. The mycoplasma in all cell lines was tested using PCR gel electrophoresis. 1: Positive control; 2: HNBE; 3: NCI-H1299: 4: NCI-H1395; 5: A549; 6: NCL-H46.

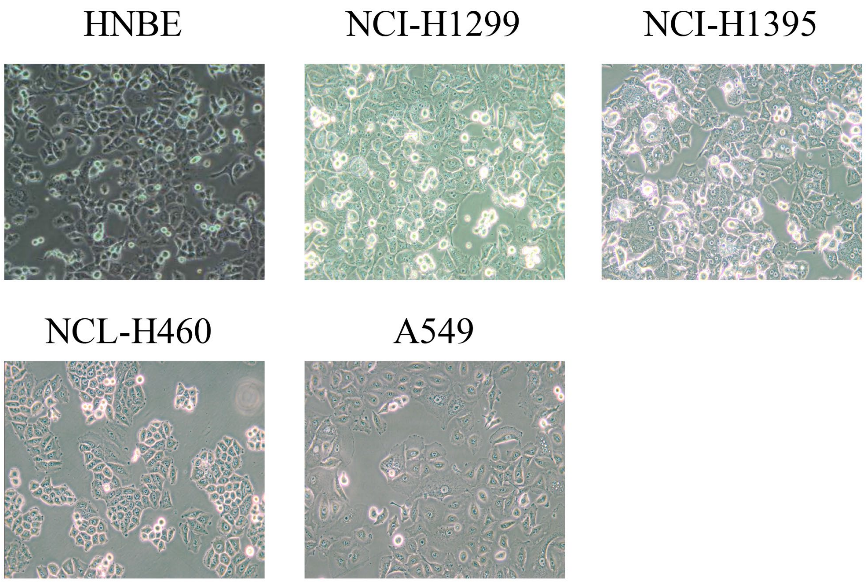

Supplementary Figure 2. Morphology images of cell lines.
